# Impact of Think-Aloud on Eye-Tracking: A Comparison of Concurrent and Retrospective Think-Aloud for Research on Decision-Making in the Game Environment

**DOI:** 10.3390/s20102750

**Published:** 2020-05-12

**Authors:** Michal Prokop, Ladislav Pilař, Ivana Tichá

**Affiliations:** Department of Management, Faculty of Economics and Management, Czech University of Life Sciences Prague, 165 21 Prague, Czech Republic; pilarl@pef.czu.cz (L.P.); ticha@pef.czu.cz (I.T.)

**Keywords:** decision-making, eye-tracking, games, simulation game, think-aloud

## Abstract

Simulations and games bring the possibility to research complex processes of managerial decision-making. However, this modern field requires adequate methodological procedures. Many authors recommend the use of a combination of concurrent think-aloud (CTA) or retrospective think-aloud (RTA) with eye-tracking to investigate cognitive processes such as decision-making. Nevertheless, previous studies have little or no consideration of the possible differential impact of both think-aloud methods on data provided by eye-tracking. Therefore, the main aim of this study is to compare and assess if and how these methods differ in terms of their impact on eye-tracking. The experiment was conducted for this purpose. Participants were 14 managers who played a specific simulation game with CTA use and 17 managers who played the same game with RTA use. The results empirically prove that CTA significantly distorts data provided by eye-tracking, whereas data gathered when RTA is used, provide independent pieces of evidence about the participants’ behavior. These findings suggest that RTA is more suitable for combined use with eye-tracking for the purpose of the research of decision-making in the game environment.

## 1. Introduction

Nowadays, managers face challenging conditions, dynamic environments, and complex processes [1,2]. Their decision skills affect the success of the companies [3,4] and determines their economic results [5]. Although human decision-making is described by a number of theories, it still remains a complex process that is difficult to research [6,7,8]. Moreover, to perform such research in the real world, where a significant sample of participants face identical conditions and situations, is almost impossible. Modern technologies, however, bring the possibility to use computer simulations and games for the decision-making research [1,9,10,11,12]. Games provide an adequate and safe space for experimentation [13,14,15]. Their use for the research of decision-making is based on the idea, that respondents reflect their knowledge, experience, and skills during play [11,12,16,17,18]. Nevertheless, the research of decision-making using games is still a little researched area. In addition, it requires adequate methodological procedures as different methods reveal different aspects of decision-making. Thus a description of their pros and cons will enable their better utilization [6]. In the field of decision-making research, a number of methods have been generally applied as questionnaires, observation, interviews, eye-tracking, think-aloud, decision analysis, etc. [6,19,20].

A few studies, which included the use of games, agree in particular on the use of eye-tracking [10,11]. It is the method of recording eye movements by a special apparatus (eye-tracker) [21,22]. This technique is commonly used in a human-computer interaction studies [21] and finds application in many areas of research [23]. The use of eye-tracking for research purposes is based on the assumption that humans perceive and cognitively work with what they see [24]. Vision is the most important sense in terms of acquiring information [25,26]. People perceive information by the sensory system [27] and then they integrate and compare it with expectations and knowledge, which results in a behavioral response [28]. Therefore eye-tracking is considered a valuable tool to study the cognitive processes that accompany various human mental activities, ranging from less demanding ones such as reading [26,29,30], writing [31,32,33], and perception of images and objects [34,35,36,37,38], up to the more complex ones such as learning [39,40,41,42,43,44,45], and decision-making [10,28,46,47,48]. In the case of decision-making it was proved that the method significantly contributes to the accumulation of evidence about this process [28] and improves its understanding [49]. However, there are also studies mentioning relevant shortcomings of the use of eye-tracking in decision-making research [21,47,50,51,52,53,54]. Eye-tracking cannot reveal by far all aspects of the decision-making [50], because it is not entirely clear if humans comprehend the information they watch [51], and whether watched information is incorporated into the decision-making process [52].

Therefore, the combination of eye-tracking with other methods is necessary [21,47,50]. Especially a combined use of thinking-aloud and eye-tracking data can provide deeper insights into cognitive processes [53] and help to solve the limitations associated with eye-tracking [54]. Think-aloud (often also referred to as verbal protocols) is a methodology for studying behavioral and cognitive processes while people solve problems [55]. However, there are two main types (methods) of think-aloud: (1) Concurrent think-aloud (CTA), where subjects are asked to do the tasks and verbalize thoughts simultaneously; and (2) Retrospective think-aloud (RTA) where subjects are asked to do the task silently first and then retrospectively report on the solving process [56]. Over time, both methods have penetrated many fields of science, which gives rise to constant discussions of their advantages and disadvantages, as well as their applicability and validity for various research fields [53,55,57,58,59,60,61,62]. The related problem of CTA is the dual cognitive load when the thinking process and the verbalization process compete with each other [63]. Thus, the cognitive workload of respondents can be too high [62] and it can impact on their standard working process [56]. Therefore, some ideas may be lost as they cannot be expressed in real-time [63]. The data omissions occur especially when the presented information is difficult to verbalize or the processes are automatic for participants [55,56,64,65]. RTA use does not disturb participants during the task [56,66]. Nevertheless, it has also limitations like forgetting information or omitting its interpretation [55,56,58,67] and post-rationalization or fabrication of thoughts [68,69,70]. Therefore, many scientists overcome the RTA shortcomings by re-playing recordings to participants [53,58,60,62,70].

Interestingly, many authors, who deal with think-aloud, also recommend combining them with eye-tracking [47,54,64,65,67,71]. The involvement of eye-tracking can help solve the shortcomings of think-aloud because it allows one to confirm and extend the gathered data [47] or to complete omitted and forgotten points [54].

On the other hand, these studies take little or no account of the possible impact of thinking aloud on eye-tracking data. This impact is expected specifically in the case of CTA [67,70], as this method may bring the unnatural behavior of humans during task solving [72]. Although these statements can be considered as logical, it has not been empirically verified to what extent this may be the case.

Therefore, the main aim of this study, which is focused on decision-making research in the game environment, is to compare and assess if and how CTA and RTA methods differ in terms of their impact on data provided by eye-tracking. In addition, with the respect that the game is a process, where a player approaches can evolve, the research is not only concerned with the overall final assessment and comparison of both methods but also examines whether the potential differences gradually change during the game progress or not. Based on the findings it will be possible to discuss which of the think-aloud methods in combination with eye-tracking is more suitable for decision-making process research in the game environment.

## 2. Materials and Methods

### 2.1. Simulation Game

The research was conducted through the experiment in which participants played the FactOrEasy^®^ game (FEM CZU, Prague, Czech Republic) and described their behavior using think-aloud methods. FactOrEasy^®^ is a simulation game of decision-making in financial, operational or strategic management. The user runs a virtual enterprise there and makes three mandatory decisions (Material Purchase, Production, Product Sale), and two optional decisions (Production Expansion, Taking Loan) in each round. The simulation consists of 12 rounds unless it ends earlier due to bankruptcy. The game goal is to achieve maximum “net cash” at the end of the game and beat three virtual competitors [73]. FactOrEasy^®^ is used in the teaching of managerial decision-making [73] and previous studies with managers [74] or farmers [11] proved that the game is also an appropriate tool for testing managerial decision-making skills.

### 2.2. Research Group

Thirty four (34) participants attended the experiment, each one individually. This number of participants corresponds to previous studies, which deal with think-aloud [53,57,60,61,68], eye-tracking [10,28,37,41,54,75,76], or with a combination of both [64,65,70,71]. The condition for participation was that the participant must be a manager, or business owner who has an active managerial role in their business. All subjects gave their informed consent for inclusion before they participated in the study. The study was conducted in accordance with the Declaration of Helsinki, and the protocol was approved by the Ethics Committee of CZU Prague University.

The research was conducted in two periods. In the 1st, an experiment was performed using CTA (16 participants) in the 2nd RTA took place (18 participants). Participants were randomly assigned to both groups. Each one participated only once, either in the RTA group or in the CTA group (between-subjects study design). The results of three managers have been excluded from further analysis. Two showed exceedingly poor quality of the understanding of the given task (game rules and method used). One has been excluded because of eye-tracker failure. After that eliminations, the CTA group consisted of 14 managers (nine men, five women, average age 34.8 years), the RTA group consisted of 17 players (14 men, three women, average age 30.7 years).

### 2.3. Course of Experiment

The experiment was performed in the lab of human behavior research (HUBRU) at CZU Prague University. Participants received an e-mail with links to a video tutorial and demo version of the game before the official start. They had three attempts to try the game on their own. When they arrived, the researcher firstly verified their understanding of the game and provided clarification when necessary. The instructions included only information about rules and control elements, but no advice on the procedure or strategies in the game.

After that, the researcher proceeded to explain the requirements for think-aloud in line with the standards described by Ericsson and Simon [56]. However, the RTA and CTA had to be adjusted for use in the game and for the purpose of the present study. Adjusting is nothing uncommon, and many scientists in the past had to redesign or modify these methods for their research purposes [77]. In addition, some flexibility in the process of data collection is inevitable in the methodological approaches that are used to study decision-making [6].

CTA adaptation consisted only of the prompts when the player pauses verbalization. Ericsson and Simon [56] recommend prompts after 10-15 s of silence. Most authors used reminders after just 5 s [53,64,71], but some used longer gaps [57] or did not explicitly state the time used [60]. The form of alerts used in this study is closest to Cotton and Gresty [69], who used prompts when needed, without any time fixation. We decided to alert the participants to pauses and asked them for improvement in the next verbalization always at the end of the game rounds. The aim was to do not disturb the gaming process and to avoid distorting eye-tracking metrics that could be affected during communication with the researcher.

Before applying RTA, it was necessary to consider the time needed to complete the full game (12 laps), which is usually in the range of 20–40 min [11,74]. In such a case, the participant’s memory cannot include the evidences of individual decisions in each round, even with the possible use of playback of recordings. This presumption was verified on two pilot participants (outside the research set), who were able to describe only the general strategies and procedures. In this respect, comparing CTA and RTA would not make sense, as they would provide significantly different data. Therefore, it was decided to ask participants to verbalize at the end of each round, after they finished all decisions in the given round. Following this, it was refrained from replaying the recordings to participants. This would mean stopping the game after each round and performing this action. The estimated time demands and negative impact on the gaming process were considered as inadmissible. However, the game itself allows participants to recall their previous steps. Until the player clicks the “next round” button, all figures from the given round remain on the screen. These numbers imply the results of the player’s decisions and thus can remind their circumstances. This fact was explained to RTA’s participants. With this measure, it is possible to provide a higher number of verbalizations received by the RTA in line with Olsen et al. [67] assertion that any reminder of own actions is better for participants than none. After explaining the use of CTA and RTA, the researcher verified their understanding by a few simple mathematical tasks in Microsoft Excel (Microsoft Corporation, Redmond, WA, USA).

The last step before the game started was to set up an eye-tracking. The lab is equipped with a Tobii X2-60 Eye Tracker (Tobii Pro AB, Stockholm, Sweden) and Tobii Pro Studio 3.4.8. software. The eye-tracker was situated under the 24” monitor (1920 × 1080 px, 60 Hz). “Screen record” as working mode and “I-VT” as fixation filter were set in the software. 5-point calibration was performed. The participants were seated approximately 60 cm away from the screen. They were also asked to keep their heads as still as possible in the same way to minimize inaccuracy caused by head movements.

After explaining all experiment procedures and setting up eye-tracking, the researcher left the room. Participants were observed from the control room. Mutual communication was possible using loudspeakers and microphones but was limited only to situations when the participant asked for a rule to be repeated; when the verbalization stopped (prompts) and/or in moments between two games (request for a break). Video, eye-tracking and audio records were recorded from all sessions. The number of game attempts was not limited but the length of one session (including welcome, initial briefing, equipment settings, and breaks) was limited to two hours.

### 2.4. Data Processing

#### 2.4.1. Comparison of Game Results Achieved by RTA and CTA Players

Several studies [10,39,40] claim a significant difference in data gathered by eye-tracking when a monitored task is performed by participants with different levels of skills, experience, or education. To play FactOrEasy^®^ represents a complex task that may reflect the decision-making skills of players [11,74,78]. We selected a relatively homogeneous group of participants for the experiment and distributed them evenly into CTA and RTA groups. Even so, both groups of players could have shown significantly different performances in the game. This would mean that eye-tracking metrics might have been affected not only by the use of CTA and RTA methods but also by the ability of participants to solve a given task (to play the game). In such a case, it would be very difficult to separate the two mentioned influences and draw conclusions about the different impact of CTA and RTA on the data provided by eye-tracking. Therefore, it was first necessary to assess whether the performance of players among groups did not differ significantly.

We assume that the performance of players, and thus their decision-making skills, can be indirectly evaluated using the results they have achieved in the game [74,78]. Thus, we used the analysis of the game results to compare the players’ performance of both groups. However, the simulation is stochastic, which means that even if a player faces repeating actions, their conditions may be different [73]. In order to make the comparison of players’ performance more independent of the stochastic nature of the game, we decided to compare three different indicators:

(1) The amount of “net cash”, which maximization is the main game goal. Nevertheless, this indicator cannot be calculated in bankrupt games. Therefore, it includes only completed games.
(2) The comparison of the numbers of bankruptcy games is presented as a separate indicator.
(3) The overall standings of the player at the end of the game. To beat three competitors is the second game goal. However, players with different strategies can reach different but still the highest net cash [73,78]. Therefore, the overall standings, as the indicator, is less dependent on stochastic game conditions.

The differences between indicators were statistically verified. In the case of net cash and the overall standings, the significance of the differences was tested using t-test and/or Mann-Whitney test. The test choice was based on the validation of data normality (Shapiro-Wilk). The numbers of bankruptcy games are expressed absolutely as their count within both groups, and relatively (%) in the form of the proportion of bankruptcy games to the total number of game attempts of each group. The difference was tested by the Z-test for two proportions.

#### 2.4.2. Comparison of the Impact of CTA and RTA on Eye-Tracking

In order to compare the impact of CTA and RTA on eye-tracking, it was first necessary to choose time segments of gathered records, which would be appropriate for comparison of eye-tracking metrics. One game attempt is not a suitable segment due to their low gathered number (2.32 on average per player). This option wouldn’t allow a more detailed comparison of metrics’ development over time. One game round seemed a better choice (22.45 on average per player) but is not suitable for analysis as a whole due to two assumptions. The 1st is the abovementioned modification of CTA. Although prompts were realized at the end of rounds, they were still part of eye-tracking records. In such cases, the records are influenced by communications with the researcher. These moments occurred in different numbers for different players and with different duration. Therefore, it is difficult to separate and exclude them from records. The 2nd problem is that game rounds can involve a different number of decisions. Three are always mandatory, but Loan and Factory can bring a higher number of decisions in one round. Those could bring a higher number of eye-movements that cannot be excluded from records. Therefore, including the rounds with more decisions can distort results.

**Figure 1:**
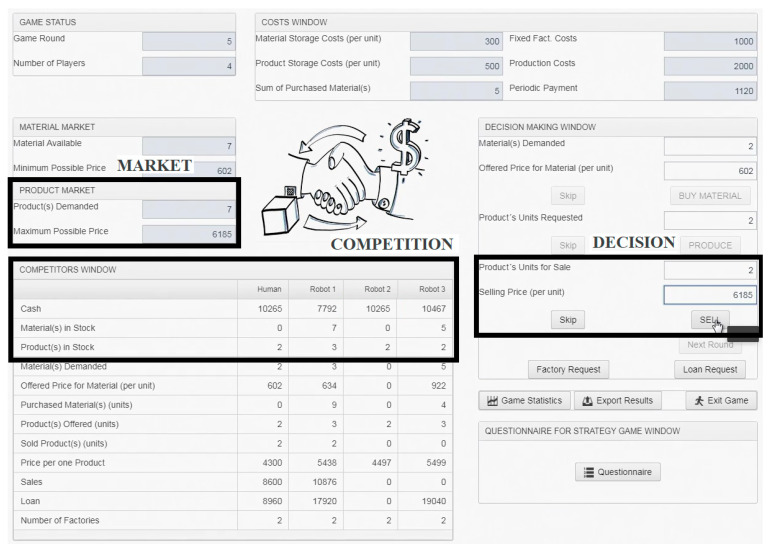
The game window of FactOrEasy^®^ with the areas of interest (AOIs), that contain figures necessary for the decision in the selling phase.

In view of the above limitations, the choice of an appropriate segment was limited to one phase of the game that is mandatory in each round. This measure provides a sufficient number of segments (same as the number of game rounds) and relatively independent eye movement data (associated with only one decision). The selling phase (Product Sale) was evaluated as the best option for this purpose due to the easy extraction of its segments from the eye-tracking records and clear rules and circumstances of this phase. The start of the segment was determined by clicking the “Produce” button (ending the production decision and the start of the selling phase) and the end by clicking the “Sell” button (which ends the phase). In this phase, the player usually offers products for sale (if he/she have some). The sale takes the form of a reverse auction. There are three important areas of interest (AOIs) (see Figure 1), which is necessary to follow to decide about the sale: (1) the market situation (Market) shows the demand; (2) the stocks of competitors (Competition), where the player should consider overall supply; and (3) the area where the player makes a decision (Decision). The selling phase is considered as the most independent of other circumstances. Product Sale is the last mandatory decision and thus it is not needed to consider future steps in the round (unlike in Material Purchase and Production). Therefore, the player should focus his/her gaze mainly on the three mentioned AOIs since the other areas on screen are irrelevant to the decision. In addition, either irregular decisions should not occur during these segments, because they are also irrelevant during this game period. Finally, segments are free of CTA’s prompts and RTA’s comments because they happen, at the earliest, after clicking the “Sell” button.

334 CTA and 362 RTA segments were gathered. Nevertheless, some of them had to be excluded from further analysis. First, 37 CTA and 25 RTA segments, when the player decided to skip the decision, were excluded. This occurs when one has no products to sell. In these circumstances, the player is forced to skip the sale. This case is accompanied by a much lower number of measured phenomena, as it is not necessary to follow the mentioned AOIs. The second exclusion included nine CTA segments (from one player) and eight RTA segments (from two players: 5 and 3). In these cases, the players significantly changed the sitting position, resulting in the loss of eye-tracking records. In the end, 288 CTA’s and 329 RTA’s segments remained for the purpose of analysis.

The next issue was to select appropriate metrics for analysis as eye-tracking offers many options [43,45]. Nevertheless, all are based on two basic eye movements—saccades and fixations [22]. Therefore, the comparison of the RTA and CTA in this study starts with two elementary indicators:

- *Number of fixations.* Eye-tracking offers the possibility to measure both the number of fixations and the number of saccades, but both counts can be interpreted identically. They show the number of places viewed by the observer [75]. Nevertheless, the first indicator was preferred in this study as fixations relate to the cognitive processing of information [24], whereas the information is not acquired during saccades [29]. In the game, the players look for cues for the right decision. The number of fixations may indicate how difficult the task is for them, because, during more difficult decisions, participants tend to show a higher number of fixations [79].
- *Fixation duration*. This metric can be interpreted in two ways. The first presumption is that a longer duration means greater interest in an object. The second states that it is associated with the complexity of the cognitive processing of information. Therefore, fixation duration can be understood as an index of the cognitive effort degree needed for information processing [21,80]. The fixation duration is expressed in two ways: (1) as the *Average duration* of (one) fixation in milliseconds (ms) and (2) the *Total duration* of (all) fixations in seconds (s).

However, eye-tracking offers many other methods, metrics, and indicators. Their right choice must be always adequate to research intentions [43,45]. For the purposes of this research, we designed and added one own indicator:

- *Dwell time ratio.* The time spent within AOI is defined as one visit (duration of all fixations and saccades are counted) from entry to exit [22]. It is also called the dwell time. The count of all dwell times within one AOI is called total dwell time [21]. Thus, based on this metric, the proposed Dwell time ratio (Rdw) is defined as the ratio which includes several total dwell times:
  (1) $$Rdw=\frac{Tdw_{m}+Tdw_{c}+Tdw_{d}}{Tdw_{s}}\times 100\;\left(\%\right)$$
  where the numerator of the ratio is the sum of three total dwell times (Tdwm, Tdwc, Tdwd), which represents the sum of visits in Market, Decision, and Competition (Figure 1). The denominator includes one AOI that represents the total dwell time in the whole game window (Tdws). The ratio result then indicates the percentage of the time the players spent watching AOIs necessary to the decision. The purpose of this metric is to assess which players are better able to focus their gaze only on important AOIs.

The comparison of all eye-tracking metrics between CTA and RTA is examined from two perspectives. First is the overall comparison, which includes data from all segments within groups. Means and medians of each metric were computed and the statistical significance of the differences among groups was tested by the Mann-Whitney test, with respect, that there was no normal distribution of data within most groups (*p* < 0.05, tested by Shapiro-Wilk).

The second perspective is the comparison of metrics’ development over time—during the game progress—based on the order of rounds (segments) played. It was assumed that selected metrics may change with increasing number of finished rounds. Therefore first, for each player, the individual values of metrics were arranged in chronological order according to the order of rounds in the game. This was done regardless of what game attempt the round was part of, i.e., regardless of eventually early termination of the game due to bankruptcy (e.g., if a player went bankrupt in 8th round and started a new game, the first round of the new game was marked as 9th). Then, for both think-aloud groups, the means of the given metrics in each round were calculated. The segments which were excluded from analysis (due to skipped selling phase and missing eye-tracking data) were not included in the computation of means. Nevertheless, they were excluded only from computation, there was no influence on arranged round order. In a practical application, this means that e.g., the mean of the 4th round of CTA was calculated from the values of 14 players, the 5th round includes the only 13 values (because one player skipped the sale) and 6th round includes again 14 values (everyone solve the decision again). We assumed the arranged rounds’ order should be maintained, despite the fact of segment exclusion. In these cases, the player still gained some experience with the playing. Therefore, the continuity of progress should be maintained.

Table 1 shows the number of segments, which are included in the analysis of each round. The numbers decrease as different players have played a different number of rounds. After the 18th round, less than half of CTA’s segments are included in results. Therefore, we consider the explanatory power of data in the following rounds as low. As a result, we limited the results shown in charts to 18 rounds.

#### 2.4.3. Comparison of the Data Gathered by CTA and RTA

Although the main aim of the study is to compare the data gathered by eye-tracking, this assessment should not remain entirely isolated. To compare RTA and CTA, it is also important to consider the potential differences in comments provided by both methods. Therefore, at least a basic comparison of the data gathered by both verbalization methods should be provided.

Differences in the gathered data are usually assessed by qualitative analysis of transcripts of audio recordings. This is usually happened by coding texts according to the type of content [58,61,64,65]. Many different types of coding have been developed—either for particular environments in which they were used or according to the particular research intentions of their creators. As a result, the findings of studies across different research fields often differ significantly. Therefore, it is questionable how useful it would be to replicate some of the previous forms of qualitative coding in our specific environment. In addition, for a basic comparison of the gathered data, we consider suchlike analysis to be disproportionately extensive and extending beyond the aims of this study.

Therefore, we decided to assess the data gathered by CTA and RTA less sophisticatedly, but on the other hand, in a way that is more related to the topic of the present study. The way of our analysis is based on the study of Guan et al. [70], who used a similar approach for verification of data provided by RTA. For both methods, we examined whether participants’ explanations include information, which is necessary to consider for a decision. As in the case of the comparison of eye-tracking metrics, we limited the analysis to the selling phase. As was mentioned there are three AOIs that are necessary to follow during the making of this decision—Market, Competition, Decision. Therefore, we examined if players verbalized the content of these three AOIs when they describe their decisions. In other words, we compared which of the think-aloud methods provides more evidence about what information from the game screen were considered during decision-making. Among other things, this comparison allows us to find out how both methods are connected with the risk of the loss of such information. Based on this, it will be possible to better assess the need for a combination of CTA and/or RTA with eye-tracking.

The analysis was performed as follows. For each individual decision, it was necessary to check whether the AOIs were really watched and then to compare these findings with the data gathered by verbal protocols. Four combinations may happen:

(1) Valid information—AOI is watched and its content is verbalized
(2) Omitted information—AOI is watched but its content is not verbalized
(3) Fabricated information—this phenomenon is connected only with RTA [68,69,70], respectively is very unlikely in the case of CTA. It means that AOI was not watched but a participant talks about its content.
(4) Unidentified information—AOI is not watched and verbalized. This is, in a way, also valid information. However, it is of a different type from the first-mentioned combination. It provides evidence of the non-inclusion of AOI in the decision-making process. Therefore, these cases are included in a separate category.

Each of the 288 CTA and 329 RTA decisions was analyzed in the abovementioned way, which meant the assessment of 864 and 987 AOIs, respectively (three for each decision). The result of the assessment of each AOI was classified into one of four categories created according to defined combinations (watching vs. verbalizing). These categories were then used for comparison of the data gathered by CTA and RTA. The overall results (categories) are expressed in the form of absolute (count) and relative (%) frequencies. The statistical differences were tested by the Z-test for two proportions.

## 3. Results

### 3.1. Comparison of Game Results Achieved by RTA and CTA Players

CTA participants played 36 games (average 2.57 per player). The RTA group played 36 games too (average 2.12 per player). Table 2 gives a comparison of “net cash” achieved by players and their overall standings at the end of the game. Table 3 provides a comparison of the numbers of bankruptcy games. No statistically significant difference was found between the “net cash” (*t*_(48)_ = −0.447; *p* = 0.657), the player’s overall standing (*U* = 632; *p* = 0.844), and the number of bankruptcies (*z* = 0.512; *p* < 0.610).

### 3.2. Overall Comparison of the Impact of CTA and RTA on Eye-Tracking

Table 4 shows the overall comparison of the monitored metrics. The number of fixations and total fixation duration are higher for CTA than for RTA (*p* < 0.001). The average duration of fixation and dwell time ratio are lower for CTA than for RTA (*p* < 0.001).

### 3.3. Comparison of the Impact of CTA and RTA on Eye-Tracking over Time (in the Game Progress)

#### 3.3.1. The Number of Fixations

**Figure 2:**
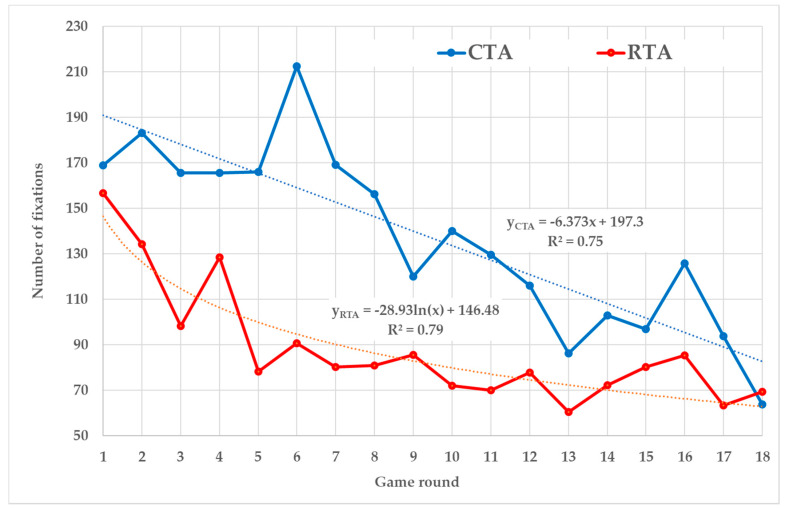
Number of fixations.

CTA players achieved more fixations than the RTA players in all rounds, except the last one (Figure 2). The CTA players’ trend is best represented by a linear line (*R*^2^ = 0.75) when the number of fixations decreases by 6.37 in each round. The RTA’s trend is described by the logarithmic function (*R*^2^ = 0.79) when the number of fixations decreases faster in the first rounds. Differences among values of both curves decrease during the game.

#### 3.3.2. Fixation Duration

**Figure 3:**
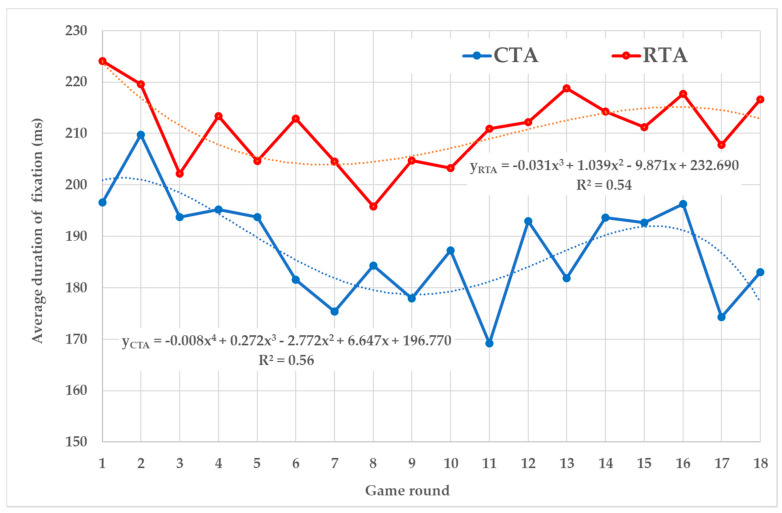
Average duration of fixation.

The average fixation duration of the CTA’s group kept lower than the RTA’s group during all rounds (Figure 3). Both trends are described by sophisticated power functions, which show that the average duration of fixation does not change distinctly during the rounds, only fluctuates within a certain range.

**Figure 4:**
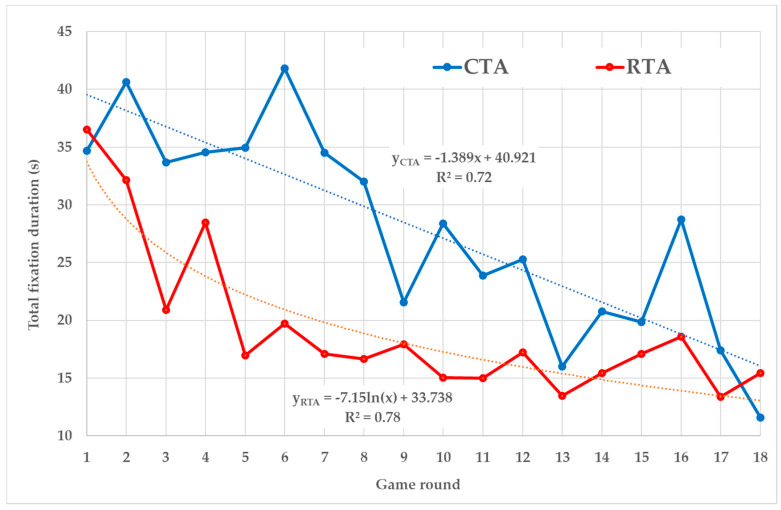
Total fixation duration.

Total fixation duration trend patterns of the CTA and RTA groups (Figure 4) are close to trend patterns of the number of fixations (Figure 2). The reason is that the total fixation duration in each round is the product of the number of fixations (which are gradually change through the rounds) and the average duration of fixation (what is the relatively unchangeable variable). The CTA group has a lower total fixation duration than the RTA group in all rounds, except the first and the last one. The CTA’s trend is represented by a linear line (*R*^2^ = 0.72) when the total fixation duration decreases by 1.38 s in each round. The RTA trend is described by the logarithmic function (*R*^2^ = 0.78) when the total fixation duration decreases faster in the first rounds.

#### 3.3.3. Dwell Time Ratio

**Figure 5:**
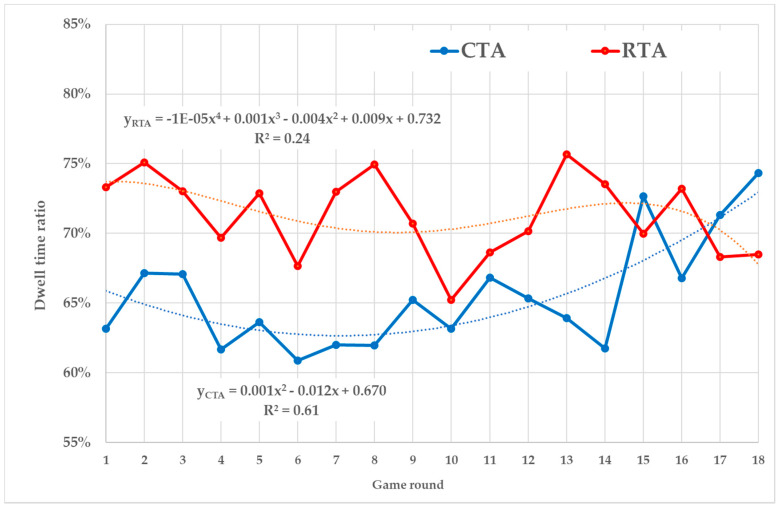
Dwell time ratio.

The progress of Dwell time ratio is shown in Figure 5. The RTA curve fluctuates greatly, and the best-found trend function does not describe its development well (*R*^2^ = 0.24). The power function of the CTA trend has a higher explanatory power (*R*^2^ = 0.61). The slight downward trend during the first rounds is alternated by the upward trend starting with the 7th round. The RTA ratio is higher than CTA up to 14th round. Then the values are equalized (the order alternates).

### 3.4. Comparison of the Data Gathered by CTA and RTA

The comparison of the data gathered by CTA and RTA is summarized in Table 5. Only four cases for CTA and four for RTA, when players did not watch some AOI, were found. In all these cases, the content of AOIs was not verbalized as well. This means both a very low proportion of unidentified information (CTA: 0.5%, RTA: 0,4%) and no occurrence of fabricated information for both think-aloud methods. No statistically significant difference was found between these two categories (unidentified information: *z* = 0.189; *p* = 0.85, fabricated information: *z* and *p* are not available).

On the other hand, a significant difference between the methods was found in the case of the count of gathered valid information (*z* = 8.988; *p* < 0.001), respectively in case of the omitted information (*z* = 9.095; *p* < 0.001). CTA’s participants verbalized the content of AOIs in 89.1% of cases, while RTA’s participants only in 72.4%. It means that RTA results in a higher share of the omitted information (27,2%) then CTA (10,4%).

## 4. Discussion

Existing studies have provided much evidence of differences between CTA and RTA. One of the frequently discussed topics is the quality and quantity of data provided by these methods [55,56,59,60,61,68]. Scientists, who have described deficiencies of verbal protocols, recommend their combination with eye-tracking, which helps to complete missing data and insights [47,54,64,65]. However, they often do not consider the possible impact of think-aloud on eye movements. In this study, it was empirically verified that there is a significant difference in data provided by eye-tracking when using CTA or RTA.

### 4.1. Overall Comparison of the Impact of CTA and RTA on Eye-Tracking

The total fixation duration is significantly higher when using CTA than when the task is carried out in silence (RTA). Longer fixation duration can indicate two types of cognitive processes: (1) an object is interesting to a participant or (2) the cognitive processing of data is more difficult [21,80]. In the case of this study, the 1st assumption can be rejected. Both groups of respondents faced the same experimental environment, the same game’s design with the same layout and data structure on the screen. There is no indication that the CTA players considered some parts of the game to be more attractive.

The 2nd assumption is much more likely. The higher total fixation duration of CTA is mainly due to their higher number because the average length of fixation is conversely a little higher for the RTA than for CTA. A higher number of fixations then also indicate a higher cognitive effort [79]. It seems that revealed higher cognitive processing is evidence of the presence of the CTA’s dual cognitive load, described by Ericsson and Simon [56]. The cognitive effort is divided among the process of task solving and the process of verbalization. Therefore, it can be considered that the number of fixations can also be divided between these processes. In other words, the part of the CTA’s fixations arises during the decision-making process itself when the player concentrates on the task, and part of the fixations arises during the verbalization when the player concentrates on speaking. The reason for this assertion is based on fact, that CTA and RTA players achieved the same game results (Table 2 and Table 3), which suggests their similar decision-making skills. Thus, it cannot be assumed that the task itself was more demanding for one or another group. Therefore, we assume, that the decision-making process itself needed the same cognitive effort, and thus also a similar number of fixations of CTA’s and RTA’s players.

The task solving process during the selling phase can be divided into several sub-processes with different demands on cognitive processing. The extent to which the fixations are divided between two cognitive processes can then be related to the complexity of these sub-processes and may vary among them. Verbalizing simple tasks carries a lower risk of the dual load than more demanding tasks [56]. Studies that investigate CTA validation by eye-tracking [65,81] suggest that for simple tasks such as reading or describing procedures, fixations take place simultaneously with verbalizations. Thus, it can be assumed that when a player reads data from the screen or performs simple tasks (mouse-clicking, decision writing), fixations may correlate to verbalizations. Contrarily, Elling et al. [64] argue that verbalization may not correlate with fixation in many cases of more demanding tasks when many verbalized thoughts cannot be associated with fixations at all. Therefore, the distribution of fixations between two cognitive processes may occur especially in cases of more complex tasks (e.g., decision-making), when participants verbalize some cognitively more demanding thoughts. In such cases, the participants performing complex tasks can also pause their speech [64,65], but this does not necessarily mean that task circumstances would not be verbalized at all. We noticed many short moments where CTA players themselves redressed the pauses. They added comments immediately after the actions during which they were silent for a few seconds. This usually happened when they needed more cognitive capacity as they thought deeply about something. This suggests that in these short periods of increased cognitive stress, players can naturally switch from CTA to RTA, by commenting on the actions, they have just completed. As a result, the number of fixations increases during these “delayed verbalizations”. The participants either observed again cues needed for the already done decision—“repeated fixations” happen, or watched some part of the screen, without a higher cognitive perception of what is observed, because they were focused only on the verbalization of previous steps—“purposeless fixations” happen. The evidence of the presence of purposeless fixations while CTA use is also provided by the Dwell time ratio, where CTA participants did more fixations outside the AOIs important for the decision.

Nevertheless, the presence and more detailed analysis of repeated and purposeless fixations should be subject to future research, as the abovementioned evidence in this study is not quite direct and unambiguous. In fact, such situations can occur in very short time periods and they may quickly alternate or partially overlap. Therefore, it may not be easy to separate the eye-tracking data accompanying individual cognitive processes when CTA is used. In addition, the negative impact on eye-tracking may not be caused by only dual cognitive load. The unnatural physical behavior of CTA participants can also contribute to the increased number of fixations as speaking may influence the head movements and thus also the eyes [67,70,72]. CTA players in the present study achieved a lower average fixation duration, but the number of fixations was much higher. This could indicate that keeping a stable gaze at an exact point of the screen was more difficult for them and a higher number of shorter fixations can occur in a certain area of this point.

### 4.2. Comparison of the Impact of CTA and RTA on Eye-Tracking over Time (in the Game Progress)

Eye-tracking data subdivided into individual game rounds offer another view on the task-solving process as well as on the verbalization process. Eye-tracking is widely used also in the education field, where several studies evidenced that there are significant differences in eye movements between experienced and less experienced participants [10,39,40]. The development of number of fixations and total fixation duration in the present study has a decreasing trend over time, which applies to the use of both think-aloud methods. As fixations indicates cognitive load [24,79], it can be assumed, that their decreasing number and total duration relates to increasing experience. Players’ performance during the game increases with experience gathered in each round. During the first rounds, players think about decisions more and therefore they paid more attention to various objects on the screen. When players’ orientation in the game and decision knowledge are getting better, then they need less time to grasp data from the screen and they are more focused only on cues necessary for the decision. Therefore, a decreasing number and total duration of fixations are evidence of the learning process within the game. The original complex and cognitively demanding processes are becoming more and more simple depending on the number of repetitions of the situation they are associated with. This is in line with the purpose of the used simulation FactOrEasy^®^, which is not only reflects the knowledge and experience of the participants [11,74,78] but also serves as a learning tool [73].

Nevertheless, the comparison of RTA and CTA curves of number of fixations (Figure 2) and total fixation duration (Figure 4) suggest that differences among the methods’ impact on eye-tracking metrics are also developed during the game progress. Higher values on the CTA curves are caused by the fact that CTA fixations are affected by both the task-solving process and the verbalization process, while the RTA curves contain the only fixations connected with task-solving. It means that eye-tracking metrics recorded in silence (when RTA is used) provide independent evidence about player’s behavior in the simulation game.

RTA’s logarithmic trends of the mentioned metrics confirm this conclusion as they correspond with the usual course of FactOrEasy^®^. In the first rounds, players try to better understand the circumstances of each decision and they set an overall strategy. This needs more cognitive effort, which is accompanied by higher values of monitored metrics. In the following rounds, players try to follow their strategies and they are more focused on individual decisions that are becoming more and more routine. This is accompanied by the lower values of monitored metrics.

In the case of CTA, it is too difficult to separate fixations associated with a task-solving process from fixations associated with verbalizations. Therefore, eye-tracking in combination with the CTA cannot be considered as accurate, for the purpose of monitoring player’s progress in the game. On the other hand, the results suggest that both cognitive processes are also undergoing certain development during the game. CTA brings higher values of the number of fixations and total fixation duration during the opening rounds than RTA. Nevertheless, the CTA values quickly decrease in the linear trend and they are getting closer to those of RTA in the latter rounds. This development allows to confirm or further extend several assumptions about CTA mentioned in the paper:

- The CTA can make participants feel unnatural and confused. It may take some time while they get used to the task, especially at the beginning of the experiment.
- Both cognitive processes become less and less demanding depending on the experience gained. As well as players repeat the process of task-solving in each round, they also repeat verbal comments on what they do. At the beginning of the game, participants must create verbal expressions, name objects and thoughts, and interpret logical connections among them. This process is optimized during the game, comments are shortened, and the verbalization becomes more natural.
- When the task-solving process is becoming easier (the original complex and cognitively demanding processes of decision-making are getting simple), participants can be better focused on verbalizations. The verbalizations of simpler tasks are more natural and therefore, they start to correlate more frequently simultaneously with eye movements.

The abovementioned findings are also supported by the results of dwell time ratio. The CTA’s trend has been increasing over the rounds and has been reaching the same values as in the RTA case in recent rounds. This indicates that CTA players are increasingly focusing only on substantial AOIs and the number of purposeless fixations is reduced over the game.

### 4.3. Comparison of the Data Gathered by CTA and RTA

The latest analysis of our study aimed to compare the data gathered by CTA and RTA. It was examined whether participants really verbalized data of the game screen, which they considered during the decision-making. In this regard, we found that RTA players omitted significantly more information (27.2%) than CTA players (10.4%). Thus, we can confirm claims of a number of scholars [55,56,58], that RTA is associated with a higher risk of forgetting information. Therefore, if researchers considering the use of RTA want to have sufficient evidence of which screen information is considered by participants during the decision-making process, then they always should combine this method with eye-tracking. In the case of CTA, the risk of losing such evidence is lower because it is more natural for participants to verbalize the information, which they can read from the screen while solving the task [64,65,81].

However, a choice of the appropriate verbal protocol may depend on the specific aspects of the decision-making which are an object of the intended research. We are aware, that there are other points of view such as references about knowledge, inner conviction, justifications of thoughts, used strategies, etc. These aspects are not empirically assessed in the present study, which is one of the main paper’s limitations. Nevertheless, several previous studies proved that explanations of these aspects are better provided by the RTA method while CTA provides mainly comments on basic actions (reading, writing) and their outcomes [61,68]. Even though an accurate qualitative analysis of CTA and RTA transcripts by text coding has not been performed, the verbalizations collected during our study appear to suggest similar findings. CTA players verbalized more screen information because they read them loudly. However, they provided subsequently fewer explanations of how they cognitively worked with them. Given that both groups have achieved similar game results, this does not necessarily mean that CTA players thought about these cues less than RTA players. A much more likely explanation is that it was difficult for them to express all thoughts when they were concurrently focused on playing the game. This is consistent with previous findings that claim that some ideas may be lost during CTA use because they cannot be expressed in real-time [82]; and that cognitive processes are quicker than verbal processes, which means that people can think more about something then they are able to concurrently express [83].

In view of the abovementioned findings, we can afford to make the recommendation that a combination of RTA with eye-tracking is a more appropriate way to study the decision-making processes in a game environment. Despite the fact RTA results in more omissions of what screen information was considered during decision-making, eye-tracking can easily add this evidence. On the other hand, CTA, unlike RTA, provides less evidence of how this information is cognitively processed, which is a shortcoming that cannot be solved by adding eye-tracking.

### 4.4. Limitations of the Study

The results of this study and their validity are limited by several factors, which indicate some other possibilities for further research. First, the experiment was conducted in the specific simulation game FactOrEasy^®^. Applying think-aloud methods in different games can produce different results of eye-tracking metrics. These results may depend on game designs and on the level of the difficulty of the tasks, which participants face. Secondly, the present study deals mainly with differences in eye-tracking metrics when combining this method with verbal protocols. Therefore, it provides only a basic assessment of data obtained by verbal protocols. Accurate examination of CTA and RTA players’ transcripts by coding of the text was not performed. However, we assume that the scope of such an analysis is beyond the aims of the present study. For instance, it is very likely that such an analysis will require the development of a specific encoding method, which would be suitable for the game environment and research purposes. Therefore, we consider this topic as an appropriate topic to create a further separate study. Thirdly, only the general use of the mentioned methods for the given purpose is described. We do not provide any conclusions about the specific decision-making processes of participants, any assessment of their decision-making skills, etc. The choice of the appropriate verbal protocol, its combination with eye-tracking, and the choice of right metrics may depend on the specific aims, which will be objects of the intended research of further studies.

## 5. Conclusions

Many authors recommend using a combination of verbal protocols and eye-tracking to investigate cognitive processes [47,54,64,65,67,71] However, so far there have been few references to a possible difference in the use of CTA and RTA in terms of their impact on data gathered by eye-tracking. In this study, we empirically verified that there is a significant difference in data provided by eye-tracking when using CTA or RTA.

Gerjets et al. [71] state that eye-tracking brings an extension of think-aloud findings by pieces of evidence of fine-grained or implicit cognitive processes. However, the findings in this study suggest that such an extension makes sense only in the case of RTA use. All examined indicators empirically confirm the claims, that RTA has no impact on eye-tracking metrics, while CTA distorts them significantly [67,70,72]. Metrics recorded in silence provide independent evidence about player progress in the game. Thus, our results suggest that RTA is more suitable for combined use with eye-tracking for the purpose of decision-making research in this environment. When using CTA, eye-tracking metrics are affected by dual cognitive load and unnatural physical behavior of participants. A combination of the task-solving process with the verbalization process brings repeated and purposeless fixations, which are redundant and distorting.

However, the development of monitored metrics over time suggests that the problem of the dual cognitive load decrease in the game progress. In later game rounds, the CTA players achieved the same number of fixations as RTA players. In addition, they were also able to concentrate equally well only on important cues. It means that if participants repeat the same or similar task multiple times, the negative effect of the dual load on CTA may decrease. This is in line with studies [56,61], which recommend performing a suitable training task before the experimental one and repeating it until participants prove sufficient ability of concurrent verbalization. However, the question remains whether this recommendation is also valid for decision-making research, especially if the participants are dealing with a series of several different decisions (like in game). Frequent repetition of the training task, which is similar to the experimental one, can distort the results of the experiment itself.

Nonetheless, even the comparison of the data gathered by both think-aloud methods suggested that a combination of RTA with eye-tracking should be a more appropriate method for the research of decision-making processes in the game environment. The reason is that CTA provides more evidence about what screen information is considered during decision-making, while RTA verbalizations contain more evidence about how this information is cognitively processed during decision-making. In the case of RTA, the omissions of “what” can be resolved by adding eye-tracking data, whereas, in the case of CTA, eye-tracking cannot help to explain missing “how”.

However, the findings of the present study do not either mean a definitive rejection of the CTA for research purposes in the field of simulations and games. The final choice of method always depends on the aims of the intended research [6,56]. The conducted research was focused only on the examination of aspects of the decision-making process in the game environment. There are other fields, like usability testing [60,62,66] or education [68,84], where CTA has added value. The gathered results suggest the potential of the application, in particular, in the second-mentioned field. The CTA is often associated with reactivity [56,66] that may have a positive impact on the learning process [85,86]. Eye-tracking metrics indicate that the learning process in a game environment may have different development depending on whether the RTA or CTA is being performed. Thus, future research could focus on whether thinking-aloud can support the learning process in the environment of simulations and games, and on what benefits CTA and RTA brings for this purpose.

## Figures and Tables

**Table 1:** The number of segments included in the analysis.

**Round**	**1**	**2**	**3**	**4**	**5**	**6**	**7**	**8**	**9**	**10**	**11**	**12**	**13**	**14**	**15**	**16**	**17**	**18**
CTA	14	14	14	14	13	14	13	14	12	13	11	12	10	10	9	8	9	7
RTA	17	17	17	16	17	16	15	15	14	14	15	12	13	15	14	11	12	12
**Round**	**19**	**20**	**21**	**22**	**23**	**24**	**25**	**26**	**27**	**28**	**29**	**30**	**31**	**32**	**33**	**34**	**35**	**36**
CTA	6	6	6	4	6	5	5	5	5	5	5	4	5	3	2	1	2	3
RTA	11	11	10	8	9	10	2	3	1	1	1	0	0	0	0	0	0	0

**Table 2:** Net cash in finished games and overall standings.

Indicator	CTA				RTA				Test of Statistical Difference
	Median	Mean	SD	Test of Normality (Shapiro-Wilk)	Median	Mean	SD	Test of Normality (Shapiro-Wilk)	
Net cash in finished games	19776	19458	10540	*W*_(24)_ = 0.952 *p* = 0.300	19032	17935	13288	*W*_(26)_ = 0.962 *p* = 0.438	t-test *t*_(48)_ = −0.447 *p* = 0.657
Overall standings	2	2.25	1.36	*W*_(36)_ = 0.730 *p* < 0.001	1.5	2.36	1.477	*W*_(36)_ = 0.730 *p* < 0.001	Mann-Whitney *U* = 632 *p* = 0.844

**Table 3:** Number of bankruptcy games.

Indicator	CTA		RTA		Test of Statistical Difference (*Z*-Test for 2 Proportions)
	Count	%	Count	%	
Bankruptcy games	12	33.32%	10	27.78%	*z* = 0.512; *p* < 0.610
Total games	36	100%	36	100%	-

**Table 4:** Overall comparison of eye-tracking metrics.

Indicator	CTA				RTA				Test of Statistical Difference (Mann-Whitney)
	Median	Mean	SD	Test of Normality (Shapiro-Wilk)	Median	Mean	SD	Test of Normality (Shapiro-Wilk)	
Number of fixations	92	128.31	116.91	*W*_(288)_ = 0.768 *p* < 0.001	64	82.29	69.68	*W*_(329)_ = 0.734 *p* < 0.001	*U* = 34823.5 *p* < 0.001
Average duration of fixation	187 ms	184 ms	44 ms	*W*_(288)_ = 0.978 *p* < 0.001	208 ms	211 ms	47 ms	*W*_(329)_ = 0.963 *p* < 0.001	*U* = 33022 *p* < 0.001
Total fixation duration	17.02 s	25.83 s	25.9 s	*W*_(288)_ = 0.773 *p* < 0.001	13.04 s	17.89 s	17.21s	*W*_(329)_ = 0.699 *p* < 0.001	*U* = 39602.5 *p* < 0.001
Dwell time ratio	0.67%	0.66%	0.16%	*W*_(288)_ = 0.992 *p* = 0.106	0.75%	0.72%	0.17%	*W*_(329)_ = 0.929 *p* < 0.001	*U* = 35326 *p* < 0.001

**Table 5:** Comparison of the data gathered by CTA and RTA.

Data Assessment	CTA		RTA		Test of Statistical Difference (Z-Test for 2 Proportion)
	Count	%	Count	%	
Valid information	770	89.1%	715	72.4%	*z* = 8.988; *p* < 0.001
Omitted information	90	10.4%	268	27.2%	*z* = 9.095; *p* < 0.001
Fabricated information	0	0%	0	0%	N/A
Unidentified information	4	0.5%	4	0.4%	*z* = 0.189; *p* = 0.85
Total	864	100%	987	100%	-
